# Protected grounds of discrimination and the risk of workplace bullying

**DOI:** 10.3389/fpsyg.2025.1488010

**Published:** 2025-04-07

**Authors:** Michael Rosander, Helge Hoel, Stefan Blomberg

**Affiliations:** ^1^Department of Behavioural Sciences and Learning, Linköping University, Linköping, Sweden; ^2^University of Manchester, Manchester, United Kingdom; ^3^Occupational and Environmental Medicine Centre, Department of Health, Medicine and Caring Sciences, Linköping University, Linköping, Sweden

**Keywords:** discrimination, minority, workplace bullying, protected grounds, nonprototypicality

## Abstract

The present study investigated the protected grounds of discrimination and the risk of exposure to workplace bullying when being in a minority at work—feeling like a minority—or merely belonging to a protected group. Further we elucidated the boundary between bullying and discrimination. Based on a social identity perspective we tested hypotheses on the risks of bullying using a probability sample of the Swedish workforce. The results showed an increased risk of person-related bullying for employees who have a protected characteristic (OR = 1.87). When also feeling like a minority the risk increased substantially (OR = 5.13). Particular high risks were found for disabled and those from an ethnic minority. The risk is not merely a structural problem affecting all where bullying is construed from being treated unfairly as part of a wider collective; the results showed an increased risk of bullying of individual targets having a protected characteristic. To alleviate this requires a comprehensive approach involving policies treating it as an organizational issue, proactively as well as having safe procedures when problems surface. Creating more well-functioning workplaces will alleviate the problem for all, although to succeed those with protected characteristics would need particular consideration.

## 1 Introduction

Being part of a minority at work is linked to increased risks of mistreatment and discrimination (Fox and Stallworth, 2005; Lewis and Gunn, 2007; Lewis et al., 2020; Rosander and Blomberg, 2022). Discrimination is defined as “any conduct based on a distinction made on grounds of natural or social categories, which have no relation either to individual capacities or merits, or to the concrete behavior of the individual person” (United Nations, 1949, p. 9). Article 21 of the Charter of Fundamental Rights of the European Union prohibits “any discrimination based on any ground such as sex, race, color, ethnic or social origin, genetic features, language, religion or belief, political or any other opinion, membership of a national minority, property, birth, disability, age or sexual orientation” (European Union, 2012, p. 400). In Sweden there are seven protected characteristics: sex, transgender identity or expression, ethnicity, religion or belief, disability, sexual orientation, and age (DO, 2022). In the present study, we refer to these as protected characteristics, and belonging to such a group as being part of a protected group. We examine how these characteristics relate to the risk of workplace bullying. While a few studies have focused on specific minority groups (e.g., Fevre et al., 2013; Hoel et al., 2022; Rosander and Blomberg, 2022), this study expands current knowledge by broadening the scope to include a wider range of characteristics and exploring the perception of minority status at work—feeling like a minority—and its impact on bullying risk. We also investigate the often blurred boundary between discrimination and bullying behaviors (Lewis et al., 2020).

Workplace bullying involves repeated, unwanted, and unreasonable actions directed at an employee over time, where the individual has difficulty defending themselves (Einarsen et al., 2020); in other words, there is a distinction between isolated acts of harassment and systematic bullying. While bullying and discrimination are distinct, the boundary between them can be blurred (Lewis et al., 2020; Di Marco et al., 2021). Bullying may involve discriminatory behavior, but if discrimination is not systematically directed at a specific person, it is not considered bullying (Einarsen et al., 2020). Pincus (1996) described three types of discrimination: individual, institutional, and structural. Institutional and structural discrimination involve policies that harm minorities as a whole, while individual discrimination focuses on actions that harm a specific minority member, whether intentional or not. Only individual discrimination can form the basis for workplace bullying. An employee may be bullied without being discriminated against, as negative behaviors directed at an individual are not discrimination per se, even if the target belongs to a protected group. All this contributes to the blurring of the two concepts. In the present study, we aim to clarify the boundary between bullying and discrimination.

## 2 Theoretical underpinning

### 2.1 A social identity perspective on mistreatment at work

There is a general increased risk of differential treatment of minorities (Allport, 1954). Regarding workplace bullying, groups such as immigrants (Rosander and Blomberg, 2022), gender minorities (Eriksen and Einarsen, 2004; Rosander et al., 2023), individuals with different sexual orientations (Hoel et al., 2022), and those with disabilities (Fevre et al., 2013) face higher risks. Leymann (1993) highlighted the vulnerability of what he referred to as” socially weaker” members, such as disabled individuals or those who differed from the majority in some way (e.g., gender, ethnicity, or religion). Social identity theory (Tajfel and Turner, 1979) may help explain this treatment, focusing on the categorization of people into “us” and “them” to maintain self-esteem.

To further understand intragroup dynamics, Turner et al. (1987) introduced self-categorization theory, which expands on social identity theory by discussing, for example, depersonalisation and group prototypes. Self-categorization occurs at different levels, from personal to social identity, with a focus on group similarities and differences. Group prototypes highlight stereotypical attributes, maximising similarities within groups and differences between them (Hogg and Terry, 2000). This process leads to depersonalization, shifting focus from individual identity to group identity, which may manifest in stereotyping, normative behavior, and ethnocentrism. The uncertainty reduction hypothesis (Hogg and Terry, 2000) suggests that people strive for a stable social identity, which promotes stronger group homogeneity, especially where ambiguity exists (Hogg and Terry, 2000).

Deviance is defined as any behavior or trait that violates group norms or differs from the group prototype (Hutchison et al., 2011). Based on Jetten and Hornsey (2014), it can be argued that ingroup members belonging to protected groups may pose a threat to group cohesion or distinctiveness. Through re-categorization, non-prototypical members may be perceived as “not us” and treated as deviants, a phenomenon known as the “black sheep effect” (Marques et al., 1988). This re-categorization process increases the likelihood of negative treatment toward perceived deviants (Marques and Paez, 1994).

### 2.2 Stereotypes and prejudice

The categorization and re-categorization of group members as outsiders deviating from the group prototype are not random. When protected grounds of discrimination are involved, categorization may be influenced by stereotypes and prejudice. People often rely on categorical information rather than individual attributes when interacting, as cognitive shortcuts (Fiske and Neuberg, 1990). Stereotypes are “exaggerated beliefs associated with a category” (Allport, 1954, p. 191). These stereotypes, particularly around gender and ethnicity, may be deeply embedded in society and persist unconsciously (Cortina, 2008). As early as the 1920s, Lippmann (1922) noted that once stereotypes are established, people focus on confirming information and disregard contradictory evidence. At work, stereotypes shape norms through jargon, jokes, and subtle discriminatory behaviors (Einarsdóttir et al., 2015), serving to rationalize behavior toward certain groups (Allport, 1954).

Prejudice can be both positive (bias) and negative, but in relation to bullying, negative prejudice is most relevant. Allport (1954) defined prejudice as a “hostile attitude toward a person who belongs to a group, simply because he belongs to that group” (p. 7). Negative attitudes (prejudice) linked to stereotypes may manifest as discrimination against individuals belonging to specific social categories.

People vary in how they are influenced by stereotypes and prejudice (Roccas and Brewer, 2002). Roccas and Brewer’s concept of social identity complexity highlights the extent to which people categorize themselves and others across multiple categories rather than as a single identity. Higher social identity complexity reduces reliance on simplistic stereotypes and prejudice. When individuals view themselves and others as multi-faceted, it becomes harder to treat someone solely based on one characteristic, such as disability or ethnicity.

Belonging to more than one protected group, such as being both disabled and an immigrant, may increase the risk of harassment (Berdahl and Moore, 2006; Shaw et al., 2011). Berdahl and Moore (2006) showed that ethnic minority women face heightened risks of both sexual and ethnic harassment. Shaw et al. (2011) found interaction effects between disability, gender, age, and ethnicity, with certain combinations, like women with ethnic minority status, being at particularly high risk. The type of disability also influenced risk, with behavioral disabilities facing more harassment than physical impairments.

Protected characteristics may differ in their visibility, and for less visible traits, the level of openness becomes significant. For instance, disabilities can be obvious, such as physical impairments, or less noticeable, like neurological conditions. Similarly, openness about sexual orientation varies. A common assumption is that greater openness increases the risk of being viewed as non-prototypical, thus raising the likelihood of negative treatment. Shaw et al. (2011) found that more visible disabilities were linked to a higher risk of harassment. However, a study by Hoel et al. (2022) on sexual orientation showed that those fully open about their identity were less likely to experience bullying compared to those less open. They argued that openness is likely based on a personal assessment of the situation, considering possible repercussions and one’s ability to manage them. Interestingly, they also reported that even if one is not open about their sexual orientation, people may assume non-heterosexuality based on stereotypes, which could increase queries and rumors, making the social category more salient and thus raising the risk of being perceived as deviant.

### 2.3 Exposure to different kinds of bullying behaviors

A wide range of negative behaviors is associated with workplace bullying, including being silenced, having one’s reputation attacked, or experiencing social isolation (Leymann, 1996; Zapf et al., 1996; Einarsen and Raknes, 1997). Today, the most common categorization distinguishes mainly between work-related and person-related negative actions (Einarsen et al., 2009).

Work-related bullying involves behaviors that undermine work performance, such as increased workload, unrealistic deadlines, or degrading tasks, such as being assigned work below one’s competence (Rosander et al., 2024). Person-related bullying targets personal integrity, including offensive remarks, gossip, or social exclusion. Most people exposed to bullying experience both types (Rosander and Nielsen, 2023), but minority groups appear more vulnerable to person-related bullying (Rosander and Blomberg, 2022). Similar findings have been reported for ethnic minorities, including social exclusion (Bergbom et al., 2015) and ‘personalized bullying’ (Lewis and Gunn, 2007). Fevre et al. (2013) found that employees with disabilities face higher risks of both work- and person-related bullying, with particularly increased exposure to physical violence, intimidation, and exclusion.

A higher likelihood of person-related bullying in minority groups can be explained through self-categorization theory (Turner et al., 1987), where the need for prototypical homogeneity leads to the re-categorization of non-prototypical members (Haslam et al., 1992) and the black sheep effect (Marques et al., 1988). Person-related bullying, more than work-related bullying, may distance the target from the group, reinforcing group clarity and strengthening the group prototype (Rosander and Blomberg, 2025).

*H1:* The risk of exposure to person-related bullying is greater than work-related bullying for members of protected groups.

*H2:* The risk of person-related bullying is greater for protected groups compared to the majority not part of such groups.

### 2.4 Minority stress and assimilation stress, and being accepted or merely tolerated

Belonging to a protected group may expose individuals to prejudice and stigma, creating a stressful social environment at work, known as minority stress (Meyer, 2003). This stress is often chronic and stems from social processes, leading to expectations of stigma, rejection, and exclusion. In addition to exclusion, minority members may also feel pressure to conform to group norms, a phenomenon we refer to as assimilation stress, which describes the strain experienced when individuals feel compelled to adapt to the majority’s expectations and behaviors in order to be accepted. Already in the 1940s, Saenger (1940) described how some minority members work harder to fit in and gain the same security as majority members. Assimilation stress may be seen as indirect differential treatment, where minorities feel compelled to adhere more strongly to group norms to be accepted.

A distinction exists between being accepted and merely tolerated (Adelman et al., 2023). When minority group members are only tolerated, but not fully accepted, they may still be seen as outsiders, leading to feelings of inferiority and hindering their basic need for belonging (Baumeister and Leary, 1995; Astrauskaite et al., 2014). This distinction highlights the difference between legally belonging to a protected group and actually feeling like a minority, which may further increase the risk of bullying. Thus, we hypothesize:

*H3:* The perception of being in a minority position—feeling like a minority—when belonging to a protected group is associated with a greater risk of exposure to person-related bullying compared to the majority not part of a protected group.

*H4:* There is an increased risk of person-related bullying for those who belong to a protected group, even if they do not feel like a minority, compared to the majority not part of a protected group.

The blurring of discrimination and bullying (Lewis et al., 2020) means that experiences of discrimination may also encompass bullying, and perceptions of bullying may reflect broader discrimination. Some evidence suggests that belonging to a protected group does not always result in attributing mistreatment to discrimination. For example, Fevre et al. (2013) found that employees with disabilities, like non-disabled individuals, often attributed mistreatment to the work environment rather than discrimination. This raises questions about whether mistreatment is perceived as bullying or discrimination. Thus, we hypothesize:

*H5:* There is an increased risk of person-related bullying for those who (a) belong to a protected group, (b) belong to a protected group and feel like a minority at work, and (c) merely have a protected characteristic under discrimination law (i.e., not feeling like a minority) compared to the majority not part of a protected group—even after adjusting for a perception of being discriminated against.

Hypotheses 1–5 focus on protected groups without distinguishing between specific characteristics. Previous research indicates an increased risk of bullying for ethnic minorities, gender minorities, disabled individuals, and non-heterosexual employees (Eriksen and Einarsen, 2004; Fevre et al., 2013; Hoel et al., 2022; Rosander and Blomberg, 2022). For instance, Leymann (1993) reported a fivefold risk for employees with disabilities, Eriksen and Einarsen (2004) and Rosander et al. (2023) reported a doubled risk for male gender minorities, and Rosander and Blomberg (2022) found a doubled to fourfold risk for immigrants, depending on their place of birth. Hoel et al. (2022) reported a more than threefold risk for non-heterosexual employees. Thus, we hypothesize:

*H6:* There is an increased risk of person-related bullying connected to belonging to each of the protected groups investigated in the present study.

## 3 Materials and methods

The study is based on a probability sample of the Swedish workforce, covering individuals aged 18–65 working at workplaces with at least ten employees. Two data collections were conducted 18 months apart, but the items on protected characteristics were introduced in the second survey in spring 2019, which provides the data for this study. All participants from the first survey in autumn 2017 were invited to the second, with a response rate of 59% (*n* = 1095). Sampling and distribution were handled by Statistics Sweden (a government agency). Participants were given sufficient information for informed consent. Since all contact information was handled by Statistics Sweden, and participants were identified by codes, to us the data was anonymized from the start. The project was approved by the Regional Ethical Review Board at Linköping University (Protocol number: 2017/336–32).

### 3.1 Context of the study

The study was conducted in Sweden, where strong laws and regulations protect employees belonging to protected groups, in line with EU legislation. The Discrimination Ombudsman (DO, 2022), a governmental agency established in 2009, promotes equal rights and combats discrimination. Before 2009, separate agencies existed for equality, ethnic discrimination, disability, and sexual orientation. However, like most EU countries, Sweden does not commonly collect equality data at the workplace level that would identify differential treatment (DO, 2023).

### 3.2 Participants

The sample consisted of 42% men and 58% women (biological sex, obtained from the Swedish population register). The mean age was 51.29 years (SD = 10.05), and the average tenure at their current workplace was 14.01 years (SD = 11.69). Fourteen percent held managerial positions, and 97% had a fixed contract. Most participants (53%) had some form of university or college education, 40% had 11–12 years of schooling, and 7% had 10 or fewer years of education. Regarding minority positions, 2.5% identified as part of a sexual identity minority, 3.8% reported having a disability, 2.3% belonged to a religious minority, 5.9% identified as part of an ethnic minority, and 9% identified as part of a gender minority.

### 3.3 Measures

#### 3.3.1 Workplace bullying

Workplace bullying was assessed using the Negative Acts Questionnaire–Revised (NAQ–R, Einarsen et al., 2009), validated in a Swedish context (Rosander et al., 2024). The NAQ-R consists of 22 negative and unwanted behaviors, including work-related and person-related behaviors, that may constitute bullying if experienced systematically over time. Respondents rated how often they were exposed to each behavior in the past six months on a five-point scale, from *never* to *daily*. The present study focused on two types of bullying behaviors: work-related (WRB) and person-related (PRB) (Einarsen and Raknes, 1997). Examples of WRB include “Someone withholding information which affects your performance” and “Excessive monitoring of your work,” with a Cronbach’s alpha of 0.80. PRB examples include “Being ignored or excluded” and “Being the subject of excessive teasing and sarcasm,” with an alpha of 0.90. A cut-off score of 15 or higher was used to classify WRB and PRB, in line with the official Swedish version of the NAQ-R (Rosander et al., 2024).

#### 3.3.2 Protected grounds of discrimination

Belonging to a protected group was measured with five questions: (a) “I have a disability,” (b) “I have a religious belief different from most others at my workplace,” (c) “I am of a different ethnic origin than the majority in Sweden,” and (d) “A clear majority of people at my workplace are of a different gender than I am.” Responses were yes/no, and more than one yes-answer was possible. For sexual or gender identity, five options were provided: heterosexual, homosexual, bisexual, transgender, and ‘other’, following the prompt, “I identify myself as” (no one identified as transgender, so we will refer to this as sexual identity).

A perception of minority status at work was measured by a single question: “Based on the composition of the staff at my workplace, I feel in a minority position,” with a yes/no response. To test Hypothesis 5, we adjusted for perceived discrimination using the item: “I have felt harassed or discriminated against on the grounds of gender, sexual orientation, transgender identity or expression, age, religion or other belief, ethnicity, or disability (in the last 12 months),” rated on a five-point scale from *never* to *daily*.

#### 3.3.3 Covariates

In some analyses (Hypotheses 2–6), we adjusted for two known risk factors of workplace bullying: ambiguous roles and laissez-faire leadership (Salin and Hoel, 2020). This was done to control for the possibility that those belonging to a protected group might also work in less well-functioning workplaces compared to others, which could in itself explain a higher risk of bullying. Marginalized groups may have fewer opportunities to select their workplace (see, e.g., Carlsson and Rooth, 2007; Ahmed et al., 2013) and may face greater difficulties in securing employment in well-functioning organizations. This may be particularly relevant for individuals belonging to ethnic or religious minorities and for those with disabilities. As a result, they may be at greater risk of ending up in workplaces with weaker organizational structures.

Ambiguous roles were measured using six items from the Psychosocial Work Environment Questionnaire (Rosander and Blomberg, 2018), such as “My role, responsibilities, and tasks at work are unclear and ambiguous” (Cronbach’s alpha = 0.89). Laissez-faire leadership was measured with four reversed items from the Multifactor Leadership Questionnaire (Bass and Avolio, 1990), such as “The supervisor responds quickly when important questions need to be answered” (Cronbach’s alpha = 0.93). Both scales used a seven-point Likert scale.

### 3.4 Three categories

Participants were categorized into three groups based on their responses to the five questions about protected characteristics and the question about perceiving oneself as a minority at work: (a) protected group, (b) feeling like a minority, and (c) merely protected characteristic.

The *protected group* includes those who belong to a protected group, regardless of whether they feel like a minority at work. The *feeling like a minority* group includes individuals who belong to a protected group and perceive themselves as a minority at work. The *merely protected characteristic* group includes those who belong to a protected group but do not feel like a minority at work. The first category is overarching and includes the other two categories.

To be classified in the protected group, participants needed to answer” yes” to at least one of the five questions about protected characteristics. For the feeling like a minority group, participants needed to answer” yes” to both a protected characteristic and the minority perception question. The merely protected characteristic group consisted of participants who answered” yes” to a protected characteristic but” no” to the minority perception question.

Fourteen respondents reported feeling like a minority without indicating any protected characteristic. Since we cannot determine the basis for their perception, they were excluded from the analyses. The base category in all analyses was those who did not belong to a protected group and did not feel like a minority at work.

### 3.5 Data analysis

All analyses were conducted using Stata version 18. Logistic regression was used to test the hypotheses by calculating odds ratios (OR) for the risk of exposure in the different minority categories compared to the majority category—those not belonging to a protected group and not feeling like a minority at work. For hypothesis 1, we tested the log odds differences (following a z-distribution) between the risk of person- and work-related bullying. Except for hypothesis 1, which was tested using crude odds ratios, all other logistic regressions were adjusted for ambiguous roles and laissez-faire leadership.

## 4 Results

In total, 221 participants (20%) belonged to at least one of the protected groups investigated in the present study. Of these, 186 reported belonging to one protected group, 33 to two, and 2 to three groups. Forty-nine participants belonged to a protected group and felt like a minority at work, while 172 had a protected characteristic without feeling like a minority. Those not in a protected group had significantly lower NAQ scores, t (1079) = 4.68, *p* < 0.001, *M* = 1.18, SD = 0.25, compared to those belonging to at least one group, *M* = 1.28, SD = 0.41.

Hypothesis 1 compared the risk of person-related bullying to work-related bullying. Crude odds ratios were calculated, with the base category being those not in a protected group (*n* = 860). Participants in a protected group (*n* = 221) had over double the risk of person-related bullying, OR = 2.21, *p* < 0.001, 95% CI [1.45, 3.36], while the risk of work-related bullying was not increased, OR = 1.17, *p* = 0.488, 95% CI [0.75, 1.81]. The log odds difference was significant, *z* = 2.05, *p* = 0.040, supporting Hypothesis 1. Participants feeling like a minority at work while in a protected group had five times the risk of person-related bullying, OR = 5.00, *p* < 0.001, 95% CI [2.63, 9.50]. They also showed an increased risk for work-related bullying, OR = 2.68, *p* = 0.004, 95% CI [1.38, 5.23].

We tested Hypotheses 2–5 using six logistic regressions, adjusting for ambiguous roles and laissez-faire leadership. The base category was those not in a protected group. There was a significant risk of person-related bullying for those in a protected group, OR = 2.52, *p* < 0.001 [higher for those in two or more groups, OR = 3.62, 95% CI (1.41; 9.26)], supporting Hypothesis 2. Feeling like a minority increased the risk more than fivefold, OR = 5.13, *p* < 0.001, supporting Hypothesis 3. Merely having a protected characteristic, without feeling like a minority, still carried a significant risk, OR = 1.87, *p* = 0.023, supporting Hypothesis 4. Among participants in a protected group (*n* = 221), those feeling like a minority (*n* = 49) had a significantly higher risk of person-related bullying compared to those without such a perception (*n* = 172), OR = 2.63, *p* = 0.024, 95% CI [1.14, 6.09].

In testing Hypothesis 5, adjusting for perceived discrimination, the risk of person-related bullying remained significant for those in a protected group, OR = 1.93, *p* = 0.009, and those also feeling like a minority, OR = 3.18, *p* = 0.004, supporting Hypotheses 5a and 5b. However, the risk for those merely having a protected characteristic was no longer significant when adjusting for perceived discrimination, OR = 1.54, *p* = 0.139, meaning Hypothesis 5c was not supported. The results for Hypotheses 2–5 are presented in Table 1.

**Table 1:** Two models of risk of person-related bullying when belonging to a protected group, belonging to a protected group and feeling like a minority, and merely having a protected characteristic.

			Model 1^c^			Model 2^d^		
Variable	*n*	Cases *n* (%)	OR	95% CI	*p*	OR	95% CI	*p*
No protected characteristic	860	76 (8.8%)	1	Base		1	Base	
Protected group	221	39 (17.6%)	2.52	1.60–3.98	< 0.001	1.93	1.18–3.15	0.009
Ambiguous roles			1.59	1.33–1.89	< 0.001	1.48	1.22–1.78	< 0.001
Laissez-faire leadership			1.29	1.13–1.47	< 0.001	1.28	1.11–1.47	< 0.001
Discrimination						3.48	2.25–5.39	< 0.001
Feeling like a minority^a^	49	16 (32.6%)	5.13	2.56–10.26	< 0.001	3.18	1.46–6.95	0.004
Ambiguous roles			1.47	1.21–1.79	< 0.001	1.38	1.12–1.69	0.002
Laissez-faire leadership			1.28	1.10–1.49	0.001	1.28	1.10–1.49	0.002
Discrimination						4.04	2.38–6.87	< 0.001
Merely protected characteristic^b^	172	23 (13.4%)	1.87	1.09–3.21	0.023	1.54	0.87–2.75	0.139
Ambiguous roles			1.51	1.25–1.82	< 0.001	1.42	1.16–1.72	0.001
Laissez-faire leadership			1.36	1.18–1.56	< 0.001	1.34	1.16–1.55	< 0.001
Discrimination						3.94	2.31–6.70	< 0.001

^a^Belonging to a protected group and having a perception of being in a minority. ^b^Having a protected characteristic, but not a perception of being in a minority. ^c^Adjusted for ambiguous roles and laissez-faire leadership. ^d^Model 1 + adjusted for perceived discrimination.

As hypothesized, the risk of person-related bullying was significantly greater than work-related bullying for those in a protected group. For those in a protected group who also felt like a minority, there was a significant risk for work-related bullying, though this risk was lower than for person-related bullying. Adjusting for ambiguous roles and laissez-faire leadership, the risk for work-related bullying remained significant, OR = 2.59, *p* = 0.017, 95% CI [1.19, 5.63], but became non-significant when also adjusting for perceived discrimination.

Finally, in testing Hypothesis 6, logistic regression was conducted for each protected group: disability, ethnic minority, gender minority, religious minority, and sexual identity minority. As before, two models were tested: model 1 adjusted for ambiguous roles and laissez-faire leadership, and model 2 also adjusted for perceived discrimination. The results showed a more than sixfold risk of person-related bullying for those with a disability, OR = 6.10, *p* < 0.001, and a more than threefold risk for ethnic minorities, OR = 3.18, *p* = 0.001. However, no significant risks were found for gender, religious, or sexual identity minorities, meaning Hypothesis 6 received only partial support. The risks for disability, OR = 2.87, *p* = 0.023, and ethnic minority status, OR = 2.39, *p* = 0.026, remained significant when adjusting for perceived discrimination. All results are presented in Table 2.

**Table 2:** Two models of risk of person-related bullying when belonging to a specific protected group.

			Model 1^a^			Model 2^b^		
Variable	*n*	Cases *n* (%)	OR	95% CI	*p*	OR	95% CI	*p*
No protected ground	860	76 (8.8%)	1	Base		1	Base	
Disability	42	15 (35.7%)	6.10	2.88–12.93	< 0.001	2.87	1.16–7.11	0.023
Ethnic minority	65	13 (20.0%)	3.18	1.56–6.47	0.001	2.39	1.11–5.15	0.026
Gender minority	99	11 (11.1%)	1.47	0.73–2.97	0.278	1.32	0.63–2.73	0.461
Religious minority	25	4 (16.0%)	2.81	0.84–9.44	0.095	2.60	0.79–8.61	0.117
Sexual identity minority	27	3 (11.1%)	1.69	0.48–5.99	0.417	1.36	0.36–5.08	0.650

Each protected ground was analyzed separately. ^a^Adjusted for ambiguous roles and laissez-faire leadership. ^b^Model 1 + adjusted for perceived discrimination.

No significant risks were found for work-related bullying among any protected groups. In terms of perceived gender minority status, the risks for men and women, when analyzed separately, did not show significant differences—compared to women, the risk of bullying for men in a gender minority was OR = 2.69, *p* = 0.201, 95% CI [0.59, 12.20].

## 5 Discussion

The present study focused on the risks of exposure to workplace bullying for employees belonging to a protected group. We tested hypotheses related to types of bullying behaviors, the impact of feeling like a minority, merely having a protected characteristic, and the role of perceived discrimination. The results showed a significant risk of person-related bullying for employees belonging to at least one protected group compared to those not in a protected group. This risk was significantly greater than the risk of work-related bullying, which did not differ from that of employees not part of a protected group, supporting Hypothesis 1. The risk of person-related bullying was 2.5 times higher for those in a protected group, supporting Hypothesis 2.

As suggested, being part of a minority can result in different treatment compared to the majority, where one may be accepted or merely tolerated (Adelman et al., 2023). This distinction impacts the risk of bullying, especially when individuals are conscious of their minority status. The results revealed a fivefold risk of bullying for employees who belonged to a protected group and felt like a minority compared to the majority, supporting Hypothesis 3. Even when merely having a protected characteristic and not identifying as a minority, there was still a significant risk of person-related bullying, supporting Hypothesis 4. Comparing the two groups, those feeling like a minority had more than 2.5 times the risk of bullying compared to those merely belonging to a protected group.

Belonging to a protected group raises questions about the nature of mistreatment at work. A perception of bullying behaviors may sometimes reflect institutional or structural discrimination, where policies have harmful effects on minorities (Pincus, 1996), termed “organizational bullying” (Liefooghe and MacKenzie Davey, 2001). However, we support the view that bullying should remain an interpersonal phenomenon, separate from the impersonal aspects of discrimination (Einarsen et al., 2020). While both discrimination and bullying are harmful, organizations need distinct approaches to address each issue. Investigating pure exposure to bullying, without it being conflated with perceptions of institutional discrimination, is essential.

The results showed that even when adjusting for perceived discrimination, there remained a significant risk of bullying for those in a protected group. The risk was nearly doubled for those in a protected group and more than tripled for those also feeling like a minority at work, supporting Hypotheses 5a and 5b. However, for those merely having a protected characteristic without feeling like a minority, the risk was no longer significant after adjusting for perceived discrimination, meaning Hypothesis 5c was not supported.

Finally, we examined five specific protected characteristics separately: disability, ethnic minority, gender minority, religious minority, and sexual identity minority. The results showed an over sixfold risk of bullying for employees with a disability and a more than threefold risk for ethnic minorities. These risks remained significant even after adjusting for perceived discrimination. However, no significant risks of bullying were found for gender minorities, religious minorities, or sexual identity minorities compared to employees not in a protected group, providing only partial support for Hypothesis 6.

### 5.1 Theoretical implications

Theoretically, the present study contributes important knowledge about the risks of bullying associated with being in a workplace minority and belonging to a protected group. We found that employees in protected groups were more likely to be exposed to person-related bullying, aligning with previous studies on specific minorities, particularly ethnic minorities (Hoel and Cooper, 2000; Fox and Stallworth, 2005; Lewis and Gunn, 2007; Bergbom et al., 2015; Rosander and Blomberg, 2022). These findings can be understood through self-categorization theory (Turner et al., 1987), which suggests that groups strive for a stable and predictable social identity, especially in times of uncertainty (Hogg and Terry, 2000). A central concept here is prototypicality—defining and establishing common depersonalized attributes within the group to strengthen group cohesion. When someone deviates from the group prototype, they risk being re-categorized and treated as part of an outgroup (Haslam et al., 1992), exemplifying the black sheep effect (Marques et al., 1988; Jetten and Hornsey, 2014). Negative treatment of perceived deviants may serve to distance them from the group to preserve its prototypical homogeneity. Rosander and Blomberg (2025) suggested that the marginalizing and exclusionary nature of person-related bullying behaviors, more so than work-related bullying, contributes to this process. This explains the greater risk of person-related bullying for employees in protected groups. For the targeted individual, such treatment signals that they do not belong, compounding the stress already experienced by minority group members (Giga et al., 2008; Noon, 2018).

An interesting finding was that merely having a protected characteristic increased the risk of bullying, even when individuals did not identify as a minority at work. When individuals also felt like a minority, the risk was even higher. As Adelman et al. (2023) suggested, being merely tolerated can still lead to group processes that reject those seen as undermining group prototypicality. This effect may be heightened during times of uncertainty or high work pressure, leading to what Einarsen (1999) referred to as predatory bullying. While the risks operate at the group level, individual perceptions can vary depending on factors such as social identity complexity (Roccas and Brewer, 2002), personal tolerance for ambiguity, and situational stressors.

The perception of being a target of bullying may be influenced by a broader perception of exposure to discrimination, which may not be directed at a particular employee but stems from organizational structures or institutional practices (Pincus, 1996) that create unfair situations for members of a minority group. Recent research suggests that interpersonal discrimination, sometimes referred to as ‘modern discrimination’ (Cortina, 2008), is becoming increasingly subtle and harder to detect (Einarsdóttir et al., 2015; Di Marco et al., 2021), especially as blatant discrimination is now outlawed in many jurisdictions. However, our results clearly indicate that, even if perceptions of discrimination blur the overall experience, there is still a heightened risk of being the target of “pure” bullying if one belongs to a protected group, meaning negative behaviors systematically directed at an individual. Although discrimination and bullying behaviors may overlap, the perception of bullying is based on the target’s own understanding of the situation. This aligns with findings from Fevre et al. (2013), who showed that employees with a disability often attributed their mistreatment to the working environment rather than their disability. Nonetheless, the true cause of their exposure may stem from the perpetrators’ prejudice toward those seen as deviant because they belong to a protected group. For example, stereotypes and biases against people from specific ethnic minorities or those with disabilities may persist as remnants of discriminatory practices embedded in society (Cortina, 2008), subtly shaping social interactions and responses to workplace challenges, potentially resulting in bullying processes.

The present study focuses on being in a minority position, but it is important to note that discrimination can also occur within majority groups. For instance, women in managerial positions in female-dominated workplaces may still face discrimination compared to men in similar roles (Salin, 2001). Furthermore, employees with a protected characteristic may not always perceive themselves as being in a minority, as they may have successfully adapted to the majority’s prototypicality in terms of perceptions, values, and behavior (Hogg and Terry, 2000). As a result, they may no longer see themselves as part of a minority or be perceived as outsiders or threats by other group members.

An important aspect of the present study is that we adjusted for two known risk factors of workplace bullying: ambiguous roles and laissez-faire leadership (Salin and Hoel, 2020). Both variables were significant in all analyses, underscoring their importance in understanding workplace bullying. However, the increased risk of bullying for protected groups was not due to these factors, which might have otherwise explained the greater risk.

The clearest risk of bullying in the present study was found for employees with a disability, with more than a sixfold risk compared to those not part of a protected group. While reduced health and well-being associated with some disabilities may lead to lower tolerance for negative behavior (Nielsen and Einarsen, 2012), disabled employees may face a ‘multiple whammy’. They can be easily singled out as deviants within a workgroup, and their disability may carry its own stigma, leading to rejection and exclusion. Furthermore, as highlighted by Baillien et al.’s (2009) three-way model of bullying, an inability to perform in line with group expectations can also violate group norms, creating frustration that, when ineffectively managed, can lead to aggression and bullying. However, such explanations might also reflect widespread misconceptions about disabled individuals’ abilities and productivity (WHO and The World Bank, 2011), which can in themselves trigger bullying. Moreover, reasonable adjustments made for disabled employees, as required by anti-discrimination law, may provoke aggressive responses from colleagues who perceive these adjustments as unfair (Lewis et al., 2018), even if they are unaware of the reasons behind them. This highlights the complex social dynamics that may contribute to the higher bullying risk for disabled individuals. Importantly, this explanation does not imply a ‘blame-the-victim’ perspective but seeks to explore possible causes for the increased risk observed in the study. Additionally, we did not distinguish between visible and invisible disabilities in the present study, meaning the high risk of bullying was found despite some respondents having invisible disabilities, which might not provoke the same reactions as visible ones.

The three-way model (Baillien et al., 2009) may also apply to employees from ethnic minorities, who were found to have a more than threefold risk of bullying compared to those not in a protected group. Such responses could be driven by prejudices against ethnic minorities, particularly in terms of expectations and abilities. Alternatively, the frustration resulting from norm violations—such as perceived performance issues—could be real, potentially arising from factors like lack of training opportunities (Noon, 2007), language barriers, or other barriers to effective workplace integration. These challenges may reinforce existing prejudices and contribute to exclusionary processes, with bullying as a possible outcome.

The risks associated with ethnic identity, as well as other protected characteristics, must also be understood in the context of Sweden’s reluctance, like that of many other EU countries, to formally register protected characteristics of workers at the workplace level (DO, 2023). This reluctance may hinder efforts to systematically address inequalities and create a truly inclusive organizational culture through targeted interventions and planned activities.

The study found no significant increase in bullying risk for employees in gender minorities or sexual identity minorities. One possible explanation for this unexpected finding, particularly regarding gender minorities, is that the increased risk of bullying may only apply to men in female-dominated workplaces, as found by Rosander et al. (2023). Men in such roles may deviate more from traditional gender expectations, leading to sanctions in the form of bullying (Eagly and Wood, 1982).

While sexual orientation has been associated with increased bullying in other studies (e.g., European Union Agency for Fundamantal Rights, 2013; Hoel et al., 2022), this was not supported in the present study. Sweden’s progressive policies on LGBTQ+ rights, including the decriminalization of homosexuality in 1944 and legal protections against employment discrimination since 1987 (Government Offices of Sweden, 2018), may help explain this result.

### 5.2 Practical implications

The present study clearly demonstrated that workplace bullying is a significant issue for employees with characteristics protected under discrimination law. While some may perceive bullying as part of a broader collective experience of unfairness, there is also a genuine risk of individuals being singled out for bullying. This distinction is crucial because the strategies required to address these issues may differ. Organizations must avoid rationalizing bullying as purely institutional or structural and equally, must not reduce the problem to individual behaviors alone, as this would ignore the structural and contextual factors that perpetuate discrimination and bias (Noon, 2018). Discrimination, like other forms of mistreatment, is often ambiguous and hard to prove (Cortina, 2008). Hence, a multi-pronged approach is essential in tackling both bullying and discrimination.

Despite concerns about their effectiveness (Evesson et al., 2015), a holistic approach to bullying, particularly for employees from protected groups, should be built on strong policies and procedures. These must be treated as organizational issues, endorsed and promoted by senior management (Rayner and Lewis, 2020), and developed collaboratively with the workforce and its representatives, resulting in a strong ethical infrastructure (Einarsen et al., 2017). Also, creating conditions for a conflict management climate has been shown to be an important proactive measure, especially for ethnic minorities (Rosander and Blomberg, 2025). There is a need for policies that proactively prevent bullying and reactively address complaints (Hoel and Einarsen, 2020; MYNAK, 2022). Policies that highlight unacceptable behaviors and set clear boundaries for conduct are essential. The work environment hypothesis (Einarsen et al., 1994) suggests that bullying often stems from issues such as role ambiguity and laissez-faire leadership. Improving these aspects can create well-functioning workplaces, reducing the risk of bullying, particularly for employees with protected characteristics, as demonstrated in studies on mental health issues (Rosander, 2021).

Line managers play a pivotal role in identifying and addressing unacceptable behavior, particularly when it is directed at minority group members. These individuals may face a dilemma: either ignore the bullying and continue suffering or make a complaint and risk social exclusion (Hoel et al., 2014). Thus, organizations must ensure that complaint procedures are safe, confidential, and protect against retaliation. While anonymity cannot be guaranteed for reasons of justice, it is crucial to establish trust in the complaint process (Hoel and Einarsen, 2020).

Social identity theory (Tajfel and Turner, 1979) provides valuable insights into fostering inclusivity in the workplace. To mitigate the risk of employees with protected characteristics being viewed as non-prototypical, organizations should strive to create a more inclusive social identity, where diversity in gender, religion, ethnicity, disability, and sexual orientation is embraced as part of the group prototype. The concept of social identity complexity (Roccas and Brewer, 2002) can help reduce reliance on simplistic stereotypes, promoting the idea that individuals embody multiple social identities. For instance, a person of a different ethnic origin may also be seen as a competent colleague, a parent, or a valued member of the team. This multifaceted view can reduce the risk of bullying for those with protected characteristics.

However, building an inclusive workplace requires openness about differences. Organizations should not pretend that everyone is the same or dismiss differences as inconsequential or divisive. Addressing discrimination and fostering inclusion requires a systematic approach, identifying challenges, implementing interventions—such as positive action where necessary—and continuously monitoring and reviewing the outcomes. These efforts should span the entire employment cycle, from recruitment to exit, to ensure that the organization fosters inclusivity at every stage (Hoel and McBride, 2017).

### 5.3 Strengths and limitations

A notable strength of the present study is its use of a probability sample drawn from the entire Swedish workforce (organizations with at least ten employees). The large sample size is particularly valuable when studying minority groups and bullying, which affects a relatively small portion of employees. Another strength is that we adjusted for two known risk factors of bullying, reducing the likelihood that these factors explain the increased risks found for protected groups.

However, there are several limitations. All measures were self-reported, which may be subject to social desirability and common method variance (Podsakoff and Organ, 1986). Still, any social desirability bias likely led to underestimating, rather than overestimating, the risks. Self-reports remain the most direct way to capture subjective experiences such as bullying exposure, especially given the challenges of using alternative sources. Witness reports may be limited by difficulties in observing indirect bullying behaviors and recognising the systematic nature of exposure over time. Official records, on the other hand, typically lack information on severity, and the stigma of reporting may result in incomplete records.

We lacked information on whether disabilities were visible or not, or whether participants were open about their sexual orientation, both of which could have affected the results (cf. Shaw et al., 2011; Hoel et al., 2022). Future research should include such data.

Furthermore, a more detailed analysis of intersectionality could likely have provided a more nuanced picture of the risks of bullying [see, e.g., Berdahl and Moore (2006)]. However, our data did not allow for such analyses, as the subgroups were very small. For example, the disabled ethnic minority in our data consisted of only three individuals.

We also did not distinguish between different ethnicities based on geographical or cultural differences. All participants categorized as having a different ethnic origin than the majority in Sweden were treated as a single group, which may have led to an underestimation of the risks. Previous research has shown that individuals from countries culturally distant from Sweden face a higher risk of bullying (Rosander and Blomberg, 2022), suggesting that future research should account for this.

It is important to note that individuals experiencing bullying or discrimination may become more aware of their minority identity as they try to make sense of their experiences, potentially influencing their responses.

As a probability sample of the Swedish workforce, the data allow for some generalization of the conditions and risks in Swedish working life. However, the different social, cultural, and legal contexts in other countries may hinder broader generalization, especially to non-European countries or regions with less developed discrimination legislation. As addressed in the section about the study context, there are several aspects that make Sweden stand out in such comparisons, for example, the early establishment of a Discrimination Ombudsman.

When adjusting for perceived discrimination, we used a single item, which may be problematic in some cases. However, when an item is sufficiently narrow, straightforward, and unambiguous to respondents, a single item can provide a sufficiently good estimate of what is measured [see, e.g., Bergkvist and Rossiter (2007)]. In the case of perceived discrimination, we believe this applies.

Finally, while we used data on five protected grounds of discrimination (sex, ethnicity, religion, disability, and sexual orientation), the number of participants in religious and sexual identity minorities was small, and even fewer reported bullying. As a result, individual responses had a significant impact on the outcomes for these categories, so these findings should be interpreted with caution.

## 6 Conclusion

The present study showed an increased risk of workplace bullying for employees with characteristics protected under discrimination law. The elevated risk was significant for person-related bullying, where an individual’s integrity and standing are undermined, often leading to social exclusion. When employees feel like a minority at work—being treated as deviant—the risk increases further. However, even those merely possessing a protected characteristic without feeling like a minority still face a significant risk. This risk is not just a structural or institutional problem; it affects individual employees directly because they are categorized as belonging to a protected group.

Self-categorization theory (Turner et al., 1987) and the black sheep effect (Marques et al., 1988) help explain why minority groups are more vulnerable to bullying. Such groups are often treated as deviants, facing sanctions intended to make them conform, which can take the form of bullying behaviors. While some individuals may avoid such treatment by conforming or hiding certain characteristics, this is not always possible, especially for those with visible disabilities or from an ethnic minority.

Organizations must adopt inclusive policies that welcome diversity. A comprehensive approach to bullying—supported by policies and senior management—should treat bullying as an organizational issue. This approach should include both preventative and reactive strategies, with clear standards for acceptable behavior and attention to the experiences of minority groups creating a strong ethical infrastructure. Effective leadership and clear roles can reduce the risk of bullying, particularly for vulnerable groups. Managers bear the responsibility of addressing misconduct and ensuring a fair and safe complaints process.

To foster a more inclusive social identity within organizations, it is beneficial to recognize that individuals hold multiple social identities concurrently, as suggested by social identity complexity (Roccas and Brewer, 2002). However, there is a risk that this approach may inadvertently make social categories more salient, potentially backfiring.

Finally, distinguishing between bullying as an individual issue and discrimination as a collective experience highlights the need to address both as overlapping but separate problems. Treating bullying as an organizational problem, while promoting respect, dignity, and inclusion, will benefit all employees—especially those with protected characteristics.

## Data Availability

The raw data supporting the conclusions of this article will be made available by the authors, without undue reservation.
